# Differential Gene Expression Landscape of Co-Existing Cervical Pre-Cancer Lesions Using RNA-seq

**DOI:** 10.3389/fonc.2014.00339

**Published:** 2014-11-26

**Authors:** Kathryn E. Royse, Degui Zhi, Michael G. Conner, Buffie Clodfelder-Miller, Vinodh Srinivasasainagendra, Laura Kelly Vaughan, Christine F. Skibola, David K. Crossman, Shawn Levy, Sadeep Shrestha

**Affiliations:** ^1^Department of Epidemiology, University of Alabama at Birmingham, Birmingham, AL, USA; ^2^Department of Biostatistics, University of Alabama at Birmingham, Birmingham, AL, USA; ^3^Department of Pathology, University of Alabama at Birmingham, Birmingham, AL, USA; ^4^Cellular and Molecular Neuropathology Core, University of Alabama at Birmingham, Birmingham, AL, USA; ^5^Department of Genetics, University of Alabama at Birmingham, Birmingham, AL, USA; ^6^Hudson Alpha Institute for Biotechnology, Huntsville, AL, USA

**Keywords:** RNA-sequencing, gene expression, cervical dysplasia, co-existing lesions, human genome

## Abstract

Genetic changes occurring in different stages of pre-cancer lesions reflect causal events initiating and promoting the progression to cancer. Co-existing pre-cancerous lesions including low- and high-grade squamous intraepithelial lesion (LGSIL and HGSIL), and adjacent “normal” cervical epithelium from six formalin-fixed paraffin-embedded samples were selected. Tissues from these 18 samples were isolated using laser-capture microdissection, RNA was extracted and sequenced. RNA-sequencing generated 2.4 billion raw reads in 18 samples, of which ~50.1% mapped to known and annotated genes in the human genome. There were 40 genes up-regulated and 3 down-regulated (normal to LGSIL) in at least one-third of the sample pairs (same direction and FDR *p* < 0.05) including *S100A7* and *KLK6*. Previous studies have shown that *S110A7* and *KLK7* are up-regulated in several other cancers, whereas *CCL18, CFTR*, and *SLC6A14*, also differentially expressed in two samples, are up-regulated specifically in cervical cancer. These differentially expressed genes in normal to LGSIL progression were enriched in pathways related to epithelial cell differentiation, keratinocyte differentiation, peptidase, and extracellular activities. In progression from LGSIL to HGSIL, two genes were up-regulated and five down-regulated in at least two samples. Further investigations using co-existing samples, which account for all internal confounders, will provide insights to better understand progression of cervical pre-cancer.

## Introduction

Squamous cell carcinoma of the cervix results from a sequence of well delineated non-invasive pre-cancer stages. Based on the cytological findings of Bethesda Classification system (1), these pre-cancer stages are classified as low-grade squamous intraepithelial lesion (LGSIL), consisting of cytological atypia and histological cervical intraepithelial lesion (CIN) 1, and high-grade squamous intraepithelial lesion (HGSIL), consisting primarily of CIN 2–3 plus carcinoma *in situ* (2, 3). In 2014, it is estimated that 12,360 cases of invasive cervical cancer will occur in the United States; however, 1.25 million women are expected to be diagnosed with pre-cancer by a Papanicolaou (Pap) test (4). Similarly, ~600,000 cervical cancers are expected worldwide, with epidemic proportions of pre-cancers, mostly undiagnosed (5). Studies of cervical disease progression (6) suggest that lesions in ~60% of women with LGSIL will spontaneously regress, another 20–30% will persist unchanged, about 5–10% will progress to high-grade HGSIL, and only 1% will develop invasive carcinoma (6, 7). The likelihood of HGSIL regression is 33%; progression to invasion is >12% (6). Persistent high-risk HPV (HR-HPV) infection (8), high-viral load (9), and integration of HPV DNA (10) are likely markers or determinants of progression of pre-cancer lesions to cervical cancer; however, host factors have not been thoroughly studied. While genetic alterations in cancer are common, changes found in different grades of pre-cancer lesions are more likely to reflect causal events initiating and promoting the progression to cancer, yet little is known about these genomic changes that occur.

Some experts arguably consider that it is reasonable standard-care to follow low-grade pre-cancer lesions and HPV infections without active treatment. Since HPV infection and LGSIL are diagnosed in epidemic proportions, novel biomarkers with higher specificity for cervical lesions would improve cervical cancer screening. Better early detection biomarkers would also greatly assist in the stratification of patients for chemoprevention trials of pre-neoplasia. Currently, there are no validated diagnostic or prognostic biomarkers that identify LGSIL destined toward HGSIL or cervical cancer. Since HGSIL are near-obligate precursors of cervical cancer, it is standard clinical practice to use invasive surgical interventions to reduce the burden of progression to cancer. The detection of LGSIL that may progress to HGSIL and the prevention of this progression is an important and suitable goal for non-invasive medical intervention to reduce the incidence of cervical cancer.

Gene expression studies are quite sparse for HPV-related cervical dysplasia. Squamous cell carcinoma of the cervix results from a sequence of well delineated non-invasive pre-cancer stages. Quantifying the decisive physical changes, i.e., differential function and expression of genes in the co-existing “normal” cervical epithelium and neighboring pre-cancerous lesions; low-grade squamous intraepithelial lesions (LGSIL) and high-grade squamous intraepithelial lesions (HGSIL) will elucidate when, where, and to what extent genomic variations facilitate development and progression of pre-cancer. Since LGSIL and HGSIL lesions are generally related when they co-occur, these types of samples provide an opportunity to assess morphologic progression with regard to space and time, while controlling for internal confounders (11). Specifically, RNA sequencing (RNA-seq) can examine expression patterns of genes from formalin-fixed paraffin-embedded (FFPE) tissues, which can be used to study pre-cancer progression in co-existing LGSIL and HGSIL tissues (12). In the current study, we utilized laser-capture microdissection to extract specific tissues from co-existing neoplastic stages (normal, LGSIL, and HGSIL) on FFPE samples from six women who underwent loop electrosurgical extraction procedures (LEEP), and performed innovative RNA extraction and sequencing (RNA-seq) technologies to enable comprehensive gene expression profiling of selected cell types for comparison within and between individuals.

## Materials and Methods

### Study population and patient sample selection

**Figure 1:**
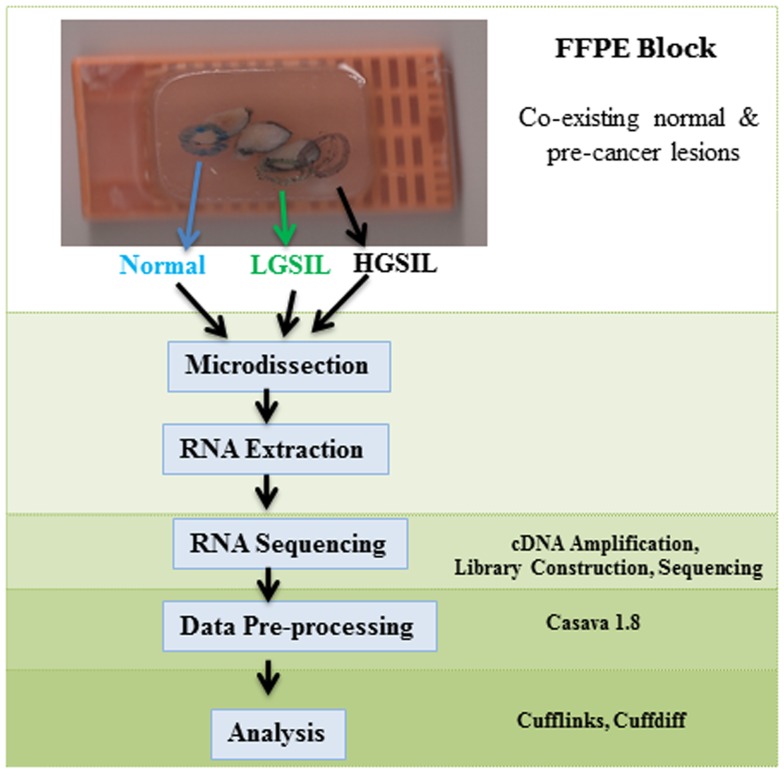
**Schematic flow of the study design, sample selection, experimental methodology, and analysis**.

The patient samples were obtained from the University of Alabama at Birmingham (UAB) Comprehensive Cancer Center (UAB-CCC) tissue procurement shared facility (TPSF) where standard protocols are followed to routinely collect cervical samples from the UAB colposcopy clinic and preserve as FFPE tissues (13). Since formalin fixation methodology, which affects nucleic acid integrity, can vary among hospital laboratories, we used only samples from women that received LEEP treatment at the UAB colposcopy clinics during June 2010 to April 2012 for abnormal cytology. A UAB pathologist prospectively reviewed cervical tissue samples from ~850 women, aged 20–25 years old to confirm if they had a co-existing spectrum of normal and pre-cancer LGSIL and HGSIL on the sample block (Figure 1). If co-existing lesions (LGSIL and HGSIL) were found for LEEP treatments during that period, women must have had HGSIL (CIN2/3) confirmed biopsies within 6 months of their treatment. Samples with evidence of immunosuppression or HIV infection were excluded due to known differences in rates of cervical abnormalities and pathogenesis (14, 15). Following these criteria, we identified 10 FFPE blocks from European American women (Figure S1 in Supplementary Material) of whom 6 were similar in relation to information on referral, cytology, demographic characteristics, and subsequent histologic biopsy (Table 1) are used for the study. Specific laboratory methods (Figure 1) were followed as specified below. The study protocols using these samples conformed to human-experimentation guidelines set forth by the United States Department of Health and Human Services and was reviewed and approved by institutional review board (IRB) at UAB.

**Table 1:** **Demographic and LEEP diagnosis information from study of cervical dysplasia FFPE co-existing HGSIL and LGSIL lesions**.

Sample ID number	Age	Smoker	HGSIL LEEP diagnosis	ICD9 code	Keratinizing dysplasia	Glandular involvement or extension	Loop margins	Adjacent normal sample location
#109	25	No	CIS of cervix uteri (includes all CIN3)	233.1	Yes	Yes	−	HGSIL
#110	23	Yes	CIS of cervix uteri (includes all CIN3)	233.1	No	No	−	HGSIL
#111	24	Yes	CIS of cervix uteri (includes all CIN3)	233.1	No	No	+	–
#113	29	Yes	Moderate dysplasia of cervix (CIN2)	622.12	No	No	+	LGSIL
#115	25	Unknown	Moderate dysplasia of cervix (CIN2)	622.12	Yes	Yes	+	LGSIL
#116	23	Unknown	CIS of cervix uteri (includes all CIN3)	233.1	No	No	−	LGSIL

### Slide preparation

Formalin-fixed paraffin-embedded blocks from six participants were used for slide preparation and microdissection. First, the pathologist, an expert in gynecologic lesion analysis, identified and marked 12 pre-cancerous lesions (LGSIL and HGSIL in each) and 6 co-existing normal tissues, respectively, to ensure the correct cell populations. Then, depending on the size of the tissues on the block, two to six 10 μm sections from the FFPE blocks were cut using RNAse-free blades at each area identified by the pathologist and mounted on the coated slide (Leica Microsystems, Buffalo Grove, IL, USA). To prevent contamination, a new blade and a different slide was prepared for each cell type (normal, LGSIL, and HGSIL) and captured separately. The samples were heated in a water bath (58°) for 2 min and the paraffin debris was carefully scraped off. No staining of samples was performed since it can further degrade RNA in FFPE tissues.

### Microdissection method

To reduce sample degradation, all laboratory procedures including sample preparation, microdissection, and RNA extraction were performed within a 24 h period. Microdissection of the 18 specific tissues (adjacent normal, LGSIL, and HGSIL) from 6 participants was performed to reduce possible contamination of different cell types in heterogeneous tissue, which could mask the detection of gene expression alterations in the cells of interest. Slide preparation, laser-capture microdissection (LCM), and RNA extraction was conducted at LifeSpan Molecular Pathology Core Services lab in Providence, Rhode Island. LCM was conducted using the Arcturus Veritas Laser-Capture Microdissection (Mountain View, CA, USA) to isolate individual cell populations from distinct normal and pre-cancer LGSIL and HGSIL cells, which were then placed on CapSure HS LCM Caps using the UV capture laser protocols. Adjacent stained slides were used as a roadmap to determine which areas would be collected. The relatively low intensity of the laser does not damage DNA, RNA, or proteins in the captured cells (16). Approximately 10,000 cells were collected per tissue sample onto a single LCM cap. Each population of captured cells was re-examined by microscopic visualization for confirmation, and before and after images of each sample were taken (Figure S1 in Supplementary Material) for additional confirmation by a gynecologic pathologist specialized in cancers.

### RNA extraction

Total RNA was extracted from all 18 microdissected tissues from 6 to 10 LCM using the Qiagen AllPrep DNA/RNA Micro Kit (Qiagen, CA, USA), according to the manufacturer’s instructions, with extended proteinase K digestion. Prior to amplification, the quality and level of degradation of the extracted RNA was assessed with RIN (RNA integrity number) assigned by the Agilent 2100 Bioanalyzer instrument using the RNA 6000 Pico kit (Agilent Technologies, Santa Clara, CA, USA). All 18 samples were stored in nuclease-free tubes and stored at −80°C until shipment for analysis.

### RNA pre-processing, library preparation, and sequencing

To increase low-RNA yields, the Nugen Ovation © FFPE RNA-Seq System (Nugen, CA, USA) was used to amplify cDNA from total RNA for transcriptome sequencing. Amplification was initiated at the 3′ end and also randomly throughout the whole transcriptome in the sample. As described in user’s manual, amplification of both mRNA and non-polyadenylated transcripts makes the Ovation RNA-Seq System (Nugen, San Carlos, CA, USA) ideal for amplification prior to next generation sequencing (NGS). The amplified cDNA was quantified using Qubit dsDNA BR Assay 2.0 (Invitrogen Life Technologies, Grand Island, NY, USA) and all samples produced ample cDNA yields (1.26–1.89 ng/μl) for library construction. Then 1 μl from each sample was diluted 5× for analysis by Agilent DNA1000 chip to verify that all profiles obtained fit NuGen’s expected profile. None of the samples were sonicated because QC analysis done after the Ovation RNAseq FFPE protocol showed that the average fragment sizes (417–466 bp) were in an acceptable range for library prep. Sequencing libraries for whole transcriptome analysis were prepared with 2 μg of each sample using Illumina Tru-Seq © Library Preparation Kits (Illumina, San Diego, CA, USA). Two 7-base index sequences were used to prepare bar-coded libraries for sample multiplexing. Two indexed libraries were loaded into each lane of flow cells. Sequencing was performed using Illumina HiSeqH2000 (Illumina, San Diego, CA, USA) at 50 base pairs, paired end, and 25 million paired reads per sample following the manufacturer’s protocol. Multiplexed single-read runs were carried out with a total of 107 cycles. The RNA-Seq dataset has been deposited in the NCBI *SRA* with accession ID SRP048735.

### Data pre-processing and alignment of sequenced reads

FASTQ sequence files were generated from the raw base-call data for all 18 samples using CASAVA 1.8 (Illumina, CA, USA), the standard data processing package from Illumina. De-multiplexing of sample indices was set with one mismatch tolerance to separate the two samples within each lane. Quality assessment of raw FASTQ reads was performed using the FASTQC program, as previously described (17).

The paired end reads were aligned against the Ensembl GRCh37.62 B (hg19) reference genome using 150 as the mate pair means inner distance and the pre-set settings in TopHat v2.0.6. The alignment quality and distribution of the reads were estimated using SAMTools v1.18 (Illumina, CA, USA). The reference genome-guided transcript assembly of the aligned reads was performed using quartile normalization, bias correction, and the default assembly settings for Cufflinks v2.1.1. The transcript abundance was calculated by estimating the fragments per kilobase of exon per million mapped fragments (FPKM), and all expressed transcripts were binned (0, <1, 1–10, and >10) on the basis of their abundance (FPKM).

### Data visualization and clustering analysis

The similarity of the relative gene transcript abundances (using Log2 transformed values of FPKM) for each of the 18 samples were compared using Spearman’s correlation calculation in SAS and an unsupervised hierarchical clustering analysis in Gene-E, a visualization tool developed by the Broad Institute (http://www.broadinstitute.org/cancer/software/GENE-E/). The pairwise comparisons of FPKM between normal to LGSIL and LGSIL to HGSIL in each sample were compared between the tissues from these three stages and visualized as stacked histograms by genomic location in each chromosome using Circos plots (18).

### Differential gene expression analysis

UCSC known genes annotation was used to assess differentially expressed genes for the pre-cancer stage effect using CuffDiff tools (19) in two different methods: (1) grouping all six samples by their tissue classification and comparing the geometric averages progressively between normal vs. LGSIL and LGSIL vs. HGSIL; and (2) comparing the geometric averages progressively within each individual separately (six pairs for normal to LGSIL and six pairs for LGSIL to HGSIL) using CuffDiff, and then merging the results across all individuals. Because of the small sample size and unique sample type, both methods were assessed to fully capture the variability in the structured experimental design. To account and adjust for multiple testing, the FDR *q*-values were calculated by CuffDiff from the raw *p*-values, which are estimated using beta negative binomial tests of variance in read counts (19). Transcripts were considered to be differentially expressed if their expression values (log2) differed by a factor of 1.5 and FDR < 0.05. For FPKM = 0, zero to positive KPKM was considered positive infinity and positive KPFM to zero was considered negative infinity (Tables S3A,B in Supplementary Material). Volcano plots of –log_10_(*p*-value) vs. Log2 (FPKM) fold-change were made to examine these association in each tissue pair within each individual.

### Pathway enrichment analysis

WEB-based GEne SeT AnaLysis Toolkit (WebGestalt) was used for gene ontology (GO) analysis to identify pathways that were enriched in all significant gene lists by each of the progression stage pairs (LGSIL vs. Normal, HGSIL vs. LGSIL) (20). Only genes that were differentially expressed in at least two participant sample comparisons (both in direction and significance at FDR *q* ≤ 0.05) were included in the analysis. Statistical significance was estimated using the hypergeometric test and GO categories were considered to be significant if the pathway included at least five genes and the adjusted *p*-value of enrichment using a Benjamini–Hochberg FDR correction was ≤0.05.

## Results

**Figure 2:**
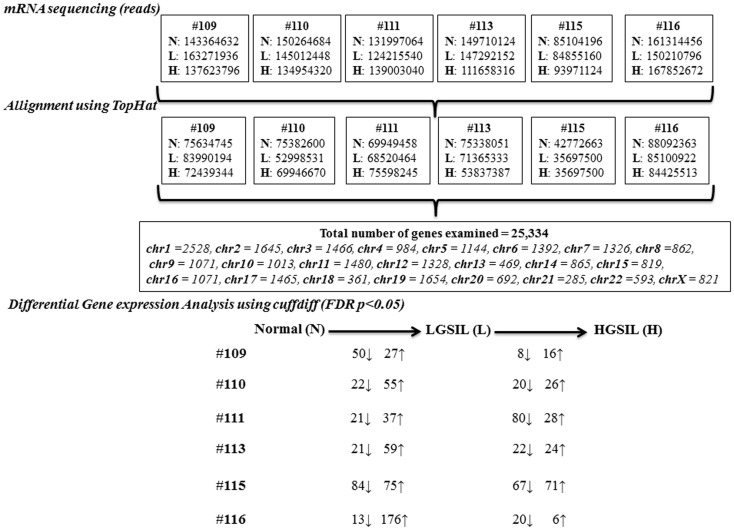
**Overview of the steps involved in the mRNA sequencing analysis of normal (N), LGSIL (L), and HGSIL (H) pre-cancer lesions in six patient samples (#109, #110, #111, #113, #115, and #116)**.

The mRNA sequencing of the co-existing spectrum of normal and pre-cancer LGSIL and HGSIL on the sample block from 6 patients (18 samples) generated a total 2.4 billion raw reads, ranging from 264 to 470 million reads per person (Figure 2; Table S1 in Supplementary Material). The overall raw read mean quality score was high (mean Phred Quality Score = 36.76) with 93.5% of bases above Q30. Among the 2.4 billion high-quality raw reads, 50.1% of the reads were mapped to the human genome with known gene annotations. Of the 29,061 genes across 22 autosomal and X chromosomes, 3727 of them had zero FPKM in all samples so 25,334 genes were examined for differential gene expression (Table S2 in Supplementary Material).

As shown in the unsupervised hierarchical clustering dendogram (Figure S2A in Supplementary Material), most of the samples from each cervical pre-cancer stage clustered together by sample and then by stage in our analysis. Sample #111 showed the most differences within the three stages and sample #115, which clustered all three stages, seemed to be in a different branch. Overall, more genes were up-regulated during progression from normal to LGSIL, with samples #115 and #116 having the largest numbers (Figure 2; Table S3A in Supplementary Material). On the other hand, more genes were down-regulated during progression from LGSIL to HGSIL, with samples #111 and #115 having the largest number of genes (Figure 2; Table S3B in Supplementary Material). Down-regulation of genes is a hallmark signature of advanced pre-cancer progression to cancer. One patient sample (#115), which consistently had a larger number of differentially expressed genes in both progression from normal to LGSIL and LGSIL to HGSIL, was one of two samples that displayed moderate dysplasia (CIN2).

None of the 25,334 genes were differentially expressed after stringent statistical correction (FDR *p* ≤ 0.05), using the first method when the analysis performed was combined for all merged samples (by pre-cancer stage) (Tables S3A,B in Supplementary Material). However, three genes (*C1orf120, EPGN*, and *NDRG4*) were differentially expressed (FDR *q* ≤ 0.20) during progression from normal to LGSIL and six genes (*ADH7, AX746562, CRNN, FABP, SLN*, and *SPRR3*) were differentially expressed (FDR *q* ≤ 0.20) during progression from LGSIL to HGSIL as listed in Table 2 (all results in Tables S3A,B in Supplementary Material).

**Table 2:** **Differentially expressed genes (FDR *q* ≤ 0.20) from transcriptome analysis of six samples combined – normal to LGSIL and LGSIL to HGSIL (Log2 transformed)**.

Gene	Locus	Normal	LGSIL	Fold-change	*p*-Value	*q*-Value
**Normal to LGSIL**						
*C1orf120*	chr1:182376755-182383948	11.0013	0	−inf	5.00E-05	0.18
*EPGN*	chr4:74979895-75196255	0	3.33617	+inf	5.00E-05	0.18
*NDRG4*	chr16:58497548-58547522	7.04797	85.1306	3.5944	5.00E-05	0.18
**LGSIL to HGSIL**						
*ADH7*	chr4:100333417-100356525	2.89046	0	−inf	5.00E-05	0.18
*AX746562*	chr7:44406738-44408891	0	4.34595	inf	5.00E-05	0.18
*CRNN*	chr1:152381718-152386750	194.411	2.41024	−6.33379	5.00E-05	0.18
*FABP4*	chr8:82390731-82395473	237.894	15.9085	−3.90245	5.00E-05	0.18
*SLN*	chr11:107578108-107582787	0	25.907	inf	5.00E-05	0.18
*SPRR3*	chr1:152974222-152976332	350.506	14.4714	−4.59817	5.00E-05	0.18

**Figure 3:**
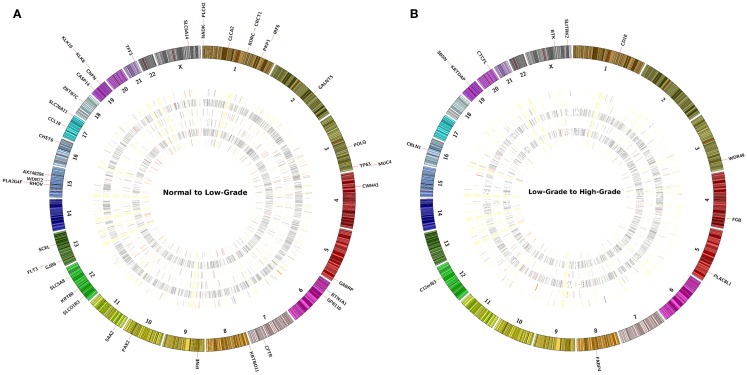
**The concentric circles represent an heatmap of −log10(*p*-value) values of pairwise comparison of the sample’s FPKM (A) normal to LGSIL and (B) LGSIL to HGSIL in each of six samples**. At the periphery of the heatmap are the genes that report a significant −log10(*p*-value) value across at least two samples or more. The concentric heatmaps are ordered from outmost to innermost in the following sample ID order: 109, 110, 111, 113, 115, and 116. Each band in the concentric circle represents a gene and its color scheme corresponds the −log(*p*-value); RED bands are values over 1.3, GRAY bands have range between 0.05 and 1.3 and WHITE (merging with the background of the plot) represents ≤0.05.

The main analysis using the second method was focused on the differentially expressed genes (FDR *p* ≤ 0.05) identified by pairwise comparison of each sample separately using CuffDiff (Figure S3 in Supplementary Material). Between 6 patient samples of normal to LGSIL pairs, there were a total of 211 genes down-regulated and 429 up-regulated (Figure 3A). Of these, *S100A7* and *KLK6* were differentially up-regulated in 3 of 6 patient samples (FDR *q* ≤ 0.05); whereas, *CCL18, FLT3*, and *RORC* were down-regulated and 38 genes were up-regulated in 2 of 6 patients (Figure 3B). In progression to high-grade pre-cancer (LGSIL to HGSIL), although there were a total of 187 different genes up-regulated in various samples, the majority (165) were present in one patient sample (#115), which displayed moderate dysplasia. Overall, *C12orf63, KRTDAP, SBSN, FABP4, CBLN1* were down-regulated whereas *CTCFL* and *PLAC8L1* were up-regulated in at least two of six samples (Table 3). When comparing the progression from LGSIL to HGSIL in these six patient samples, seven genes were differentially expressed in at least two samples (Table 3).

**Table 3:** **Differentially expressed genes in 6 pairs of normal-LGSIL-HGSIL (log2 fold-change >1.5 and FDR *p* ≤ 0.05)**.

Common pairs	Normal-to-LGSIL (*q*-value)	LGSIL-to-HGSIL (*q*-value)
3/6 Pairs	**All up-regulated**	
	*KLK6, S100A7*	
	**2 up-regulated and 1 down-regulated**	
	*ZBTB7C, GALNT5*	
2/6 Pairs	**Up-regulated**	**Up-regulated**
	*AK026736, AX748294, BTN1A1, CASP14, CFTR, CHST6, CKMT1A, CLCA2, CNFN, CRCT1, CWH43, DQ586546, GJB6, HCG22, IFNE, KBTBD11, KLK10, KLK11, KLK12, KLK13, KRT80, MUC21, NADK, PAX2, PKP1, PLA2G4F, PLCH2, POLQ, RHOV*, *SAA2, SCEL, SLC26A11, SLC5A8, SLC6A14, SLCO1B3, SPRR1B, TFF3, TP63*	*CTCFL, PLAC8L1*
	**1 up-regulated and 1 down-regulated**	**1 up-regulated and 1 down-regulated**
	*GABRP, GPR110, IRF6, LY6G6C, MUC4, WDR72*	*BTK, C9orf122, CD1E, FGB, SLITRK2, WDR49*
	**Down-regulated**	**Down-regulated**
	*CCL18, FLT3, RORC*	*C12orf63, KRTDAP, SBSN, CBLN1, FABP4*

Of the 43 differentially expressed genes in at least 2 participant samples in progression from normal to LGSIL (Table 3), 7 (*CLCA2, CRCT1, PKP1, S100A7, PLCH2, SPRR1B*, and *NADK*) were mapped to chromosome 1 and another 7 (*KLK6, CASP14, CNFN, KLK12, KLK11, KLK10, KLK13*) to chromosome 19. Cytoband location 19q13.3–q13.4 was common for differentially expressed *KLK* gene family (*KLK12, KLK11, KLK10*, and *KLK13*), the serine proteases encoded from this Kallikrein gene family have been implicated in various cancers (21). Of the 7 genes differentially expressed for LGSIL to HGSIL in at least 2/6 individuals (Table 3), *KRTDAP* and *SBSN* were mapped to chromosome 19 with the others mapped to different chromosomes.

With progression from normal to LGSIL, six genes (*SPRR1B, CNFN, TP63, PAX2, SCEL, S100A7*) were enriched in cancer pathways including GO:0030855, epithelial cell differentiation (adj *p* ≤ 7.4 E-3), and GO:0008544, epidermis development (adj *p* = 0.05). Five genes (*CNFN, SPRR1B, S100A7, SCEL, TP63*) were enriched in GO:0030216 ~ keratinocyte differentiation (*p* ≤ 1.10E-03) and GO:0009913, epidermal cell differentiation (adj *p* ≤ 1.3E-3). Several other pathways (Figure S4A in Supplementary Material) were enriched with the clusters of differentially expressed genes: GO:0017171, serine hydrolase activity (5 genes, adj *p* ≤ 1.0E-3), GO:0004252, serine-type endopeptidase activity (5 genes, adj *p* ≤ 1.0E-3), GO:0004175, endopeptidase activity (6 genes, adj *p* ≤ 1.8E-3), GO:0008509 anion transmembrane transporter activity (5 genes, adj *p* ≤ 1.8E-3), GO:0070011 peptidase activity, acting on l-amino acid peptides (6 genes, adj *p* ≤ 1.32E-2), GO:0008233 peptidase activity (6 genes, adj *p* ≤ 1.51E-2), and GO:0005576, extracellular region (13 genes, adj *p* ≤ 1.82E-2). No distinct pathways (with more than five genes and adj *p* ≤ 0.05) were enriched with genes in at least two pairs of patient samples in progression from LGSIL to HGSIL (Figure S4B in Supplementary Material).

## Discussion

We used next generation RNA-seq methods to study the transcriptomic landscapes of co-existing pre-cancer lesions and understand the mechanism of pre-cancer progression from normal to LGSIL and LGSIL to HGSIL in six individuals. To our knowledge, there have been no published reports of assessment of transcriptomic expression patterns from co-existing spectrum of neoplasia from a single sample. In most studies, samples from different individuals or from different times from the same individual are compared. A major advance in functional genomic investigations is the use of NGS, RNA-seq with *ex vivo*-derived genetic material originating from morphologically distinct cellular subpopulations within tissue. The first application combining LCM and cDNA microarray technologies to analyze gene expression in breast cancer specimens was reported in 1999 (22). Since then, most investigations of changes in gene expression associated with the progression stages in cancer have been targeted for breast cancer, prostate cancer, lung cancer, and colorectal cancer (23–30). Reports of gene expression studies of cervical cancer are sparse, specifically for pre-cancer lesions (31–33).

Using gene expression transcripts clustered by pre-cancer lesion grades, several genes were either down- or up-regulated during the progression process in each individual (Figures 3A,B). However, 43 genes were differentially expressed (in the same direction and statistically significant) in the samples from normal to LGSIL and 7 genes were differentially expressed from LGSIL to HGSIL in 2 or more individuals of the 6 participants (Table 3). In particular, *S100A7* and *KLK6* genes were differentially expressed during progression from normal tissue to LGSIL in 3/6 samples and could be key members of signature networks in cervical pre-cancer progression. The *KLK6* gene was differentially up-regulated in 3/6 LGSIL samples compared to HGSIL; whereas, *KLK10, 11, 12*, and *13* were also up-regulated in 2/6 samples. The *KLK* gene family is a member of the protease clan PA, protease family S1 with subfamily A, located on chromosome 19q13.3–q13.4. *KLK6* along with *10* and *11* and 13 are emerging biomarkers for ovarian and cancer (34). Moreover, *KLK6* has been differentially expressed in breast, uterine, and colon cancers (35–37). The *S100A7* gene, also known as psoriasin, is located within the epidermal differentiation complex on human chromosome 1q21 (38) and plays an important role as an immunomodulatory protein in skin (39). Increased *S100A7* expression has been reported in several epithelial malignancies, including head and neck squamous cell carcinoma (HNSCC) and oral dysplasia (40), which shares many features, including HPV infection, with cervical SCC, as well as skin, bladder, breast cancer, and adenocarcinomas of the stomach (41). However, we did not test for HPV and could not assess the interactions of these genes with HPV.

Three other genes are also noteworthy, chemokine (C–C motif) ligand 18 (*CCL18*) (42), cystic fibrosis transmembrane conductance regulator (*CFTR*) (43), and solute carrier family 6 member 14 (*SLC6A14*), as they have been reported to be up-regulated in cervical cancer malignancy and in the same direction as in progression from normal to LGSIL in our study (44). CCL18 is known to be involved in adaptive immune system and its role in cancer is not fully known but is best known for inducing metastasis of breast cancer cells by binding to PITPNM3 (45). CFTR, found in epithelial cells functions as a cAMP-activated ATP-gated anion channel and SLC6A14 functions as Na+ and Cl− dependent neutral and basic amino acid transporter, but their roles in cancer are not known. Of note, two genes *ZBTB7C* and *GALNT5* were differentially expressed in the same direction in two samples but opposite in the third in normal to LGSIL progression. The pathway analysis of these genes provided further insight into the expression profiling of genes involved in pre-cancer progression. The functions of the genes identified in the progression from normal to LGSIL were enriched in the biological processes and pathways of keratinocyte differentiation, epidermis development, peptidase activities, and extracellular region activities.

Inclusion of pre-cancer tissues from the same individual with respect to time and space is a major strength of this approach where all internal confounders are controlled for in determining signature gene expression patterns. However, there might be residual heterogeneity in samples between patients (Figure S2 in Supplementary Material); thus, the analysis from progression within each individual might be more informative than the combined analysis taking the mean or median values across different stages from different individuals. For instance, four patient samples had ICD9 code 233.1 (carcinoma *in situ*) (Table 1) and three of them (samples #109, #110, and #116) had *S100A7* genes up-regulated and another set (samples #110, #111, and #116) had *KLK6* up-regulated in normal to LGSIL pairs. Samples #113 and #115 may have differing cellular makeups since their HGSIL were diagnosed as moderate dysplasia (CIN2) (ICD9 code 622.12). Microdissection methods create a homogeneous sample, but any error in procedures can include adjacent heterogeneous tissues. For example, in samples #109 and #110, HGSIL cells were adjacent to normal samples (Table 1) and contaminated samples can also misclassify the expression patterns. Also, microarray technologies typically require fresh tissues; however, FFPE tissues have been regularly used in clinical research. With careful selection of samples, such as the age of the sample, length, and conditions of storage (13, 46), gene expression analyses of FFPE tissues have shown comparable results to frozen tissues (47–49). In addition, various new technologies have proven that RNA acquired by laser-capture microdissection from FFPE samples also yields reliable microarray and NGS data (50). Our use of LCM to separate a heterogeneous sample as well as presence of common gene signatures for cervical pre-cancer lead us to have more confidence in the phenotype and therefore results. Utilization of FFPE blocks in such genomic approaches is innovative as they can provide tremendous resources and opportunities for epidemiological, basic, and translational studies.

While effective prophylactic vaccines are available (for HPV types 6, 11, 16, and 18), there is no treatment for infected individuals and logistic issues of vaccine delivery still exist. Thus, discovering biomarkers that are associated with the progression of pre-cancer lesions and cancer are extremely important. We are cognizant that our study is limited by number of patient samples and our findings need to be replicated in larger study. Thus, it is unknown if some of these gene patterns observed might be rare and specific to individual patients and thus may not be used as universal biomarker for progression. However, our approach of using co-existing samples is novel and may enable accurate aggregate genomic information regarding the potential mechanism or pathway of progression, since all known and unknown confounding factors are controlled for by internal comparisons. The identified differentially expressed genes (validated in two or three samples), specifically *S100A7, KLK6, CCL18, CFTR*, and *SLC6A14*, with reports of involvement in other cancers or novel ones with potentially related biological pathways, represent targets for understanding mechanisms of pre-cancer progression.

Since complex processes regulate gene expression, it is very likely that not all important transcriptome genes were identified and conversely that some gene expression changes identified in this study may not later be confirmed, and therefore, the results of this study should be replicated in larger studies. Additionally, we chose to only assess differential gene expression in co-existing lesions although there are other processes and markers of expression including RNA-splicing and transcript expression that were not assessed in the analysis. Despite these limitations, we successfully designed and analyzed a novel application of RNA-Seq technology to identify and annotate genes and networks that may be present in cervical co-existing lesions. Future studies can examine other complexities of the transcriptome using the RNA-seq data including splice junctions, fusion, allelic variants including somatic mutations, and HPV integration that could aggregately either explain these differential gene expression patterns or progression of pre-cancer.

## Author Contributions

Sadeep Shrestha directed all aspects of the cervical pre-cancer transcriptome project. Kathryn E. Royse and Sadeep Shrestha designed the experiments and wrote the main manuscript text with help from Degui Zhi and Christine F. Skibola. Degui Zhi led the overall data QC and analyses with assistance from Kathryn E. Royse, Vinodh Srinivasasainagendra, Laura Kelly Vaughan, and David K. Crossman. Michael G. Conner helped identify the samples and pre-cancer lesions, Buffie Clodfelder-Miller provided technical assistance with microdissection protocol, and Shawn Levy directed the RNA sequencing experiments.

## Conflict of Interest Statement

The authors declare that the research was conducted in the absence of any commercial or financial relationships that could be construed as a potential conflict of interest.

## Supplementary Material

The Supplementary Material for this article can be found online at http://www.frontiersin.org/Journal/10.3389/fonc.2014.00339/abstract
