# Supplementary material for: Differential Gene Expression Landscape of Co-Existing Cervical Pre-Cancer Lesions Using RNA-seq
Source: Front Oncol. 2014 Nov 26;4:339. doi: 10.3389/fonc.2014.00339 (PMC4244708; doi:10.3389/fonc.2014.00339)

Supplementary Tables and Figures

Transcriptomic Landscape of Coexisting Cervical Pre-cancer Lesions Using RNA-seq

Kathryn E Royse[1], Degui Zhi[2], Michael G Conner[3], Buffie Clodfelder-Miller [4], Vinodh Srinivasasainagendra[2], Laura K Vaughan[2], Christine F Skibola[1], David K Crossman[5], Shawn Levy[6], Sadeep Shrestha[1*]

[1] Department of Epidemiology; [2] Department of Biostatistics, [3]Department of Pathology, [4] Cellular and Molecular Neuropathology Core,[5] Department of Genetics, University of Alabama at Birmingham, Birmingham, AL; [6] Hudson Alpha Institute for Biotechnology, Huntsville, AL

Supplementary Tables: 3
Supplementary Figures: 4

Supplementary Table S1. mRNA sequencing of FFPE Cervical Tissue Samples and their alignment statistics overview against hg19 using TopHat

Samples	Number of Reads	Total Aligned Reads (%)	Itself & Mate Mapped	Singletons
**109 HGSIL (109H)**	137623796	72439344 (52.6%)	49198336	23241008
**109 LGSIL (109L)**	163271936	83990194 (51.4%)	56203724	27786470
**109 Normal (109N)**	143364632	75634745 (52.7%)	52024314	23610431
**110 HGSIL (110H)**	134954320	69946670 (51.8%)	47067336	22879334
**110 LGSIL (110L)**	145012448	52998531 (36.5%)	21866496	31132035
**110 Normal (110N)**	150264684	75382600 (50.2%)	49699300	25683300
**111 HGSIL (111H)**	139003040	75598245 (54.4%)	53354678	22243567
**111 LGSIL (111L)**	124215540	68520464 (55.2%)	46963362	21557102
**111 Normal (111N)**	131997064	69949458 (53.0%)	47592190	22357268
**113 HGSIL (113H)**	111658316	53837387 (48.2%)	32915554	20921833
**113 LGSIL (113L)**	147292152	71365333 (48.4%)	45546810	25818523
**113 Normal (113N)**	149710124	75338051 (50.3%)	48743410	26594641
**115 HGSIL (115H)**	93971124	35697500 (34.5%)	23564902	8903745
**115 LGSIL (115L)**	84855160	35697500 (42.1%)	26715954	8981546
**115 Normal (115N)**	85104196	42772663 (50.3%)	32261016	10511647
**116 HGSIL (116H)**	167852672	84425513 (50.3%)	55190886	29234627
**116 LGSIL (116L)**	150210796	85100922 (56.7%)	60996256	24104666
**116 Normal (116N)**	161314456	88092363 (54.6%)	62469214	25623149

Supplementary Table S2. The number of 0 FPKM, low (0<FPKM<1), medium (1-10 FPKM) and high (>10 FPKM) transcripts in each FFPE cervical sample as determined by Cufflinks

Sample	0 FPKM Transcripts	Low (<1 FPKM) Transcripts	Medium (1-10 FPKM) Transcripts	High (>10 FPKM) Transcripts
**109H**	8668	5175	9508	5710
**109L**	9695	4736	8818	5812
**109N**	9283	4848	8981	5949
**110H**	9676	4508	8801	6076
**110L**	9271	4785	9316	5689
**110N**	12025	4374	6882	5780
**111H**	11806	3920	7134	6201
**111L**	10028	4261	8580	6192
**111N**	9395	4702	9013	5951
**113H**	10551	4070	8309	6131
**113L**	9367	4658	8947	6089
**113N**	10111	4363	8248	6339
**115H**	14591	4278	6190	4002
**115L**	14638	4108	6159	4156
**115N**	14091	4377	6508	4085
**116H**	8164	5418	9615	5864
**116L**	8119	5518	9702	5722
**116N**	8923	5526	8963	5649

Note: 3727 transcripts had 0 FPKM in all 18 samples

Supplementary Tables S3a & S3b (text file)

Supplementary Figure Legends

Supplementary Figure S1 | Histological reference along with before and after laser capture microdissection images of FFPE cervical tissue.

Supplementary Figure S2 | A heat map visualizing an unsupervised clustering analysis. Samples clustered by pre-cancer status (normal, LGSIL and HGSIL) and by participant (ID).

Supplementary Figure S3 | Volcano plots showing the differential expression of the transcripts ($-log_{10}$(P-value versus Log2(Fold change) between normal versus LGSIL and LGSIL versus HGSIL

Supplementary Figure S4 | The DAG diagram of the GO terms from the Biological Process enriched in the analysis using WebGestalt toolkit from differentially expressed genes in at least 2 individuals during progression from (A) normal to LGSIL and (B) LGSIL to HGSIL

Supplemantary Figure S1

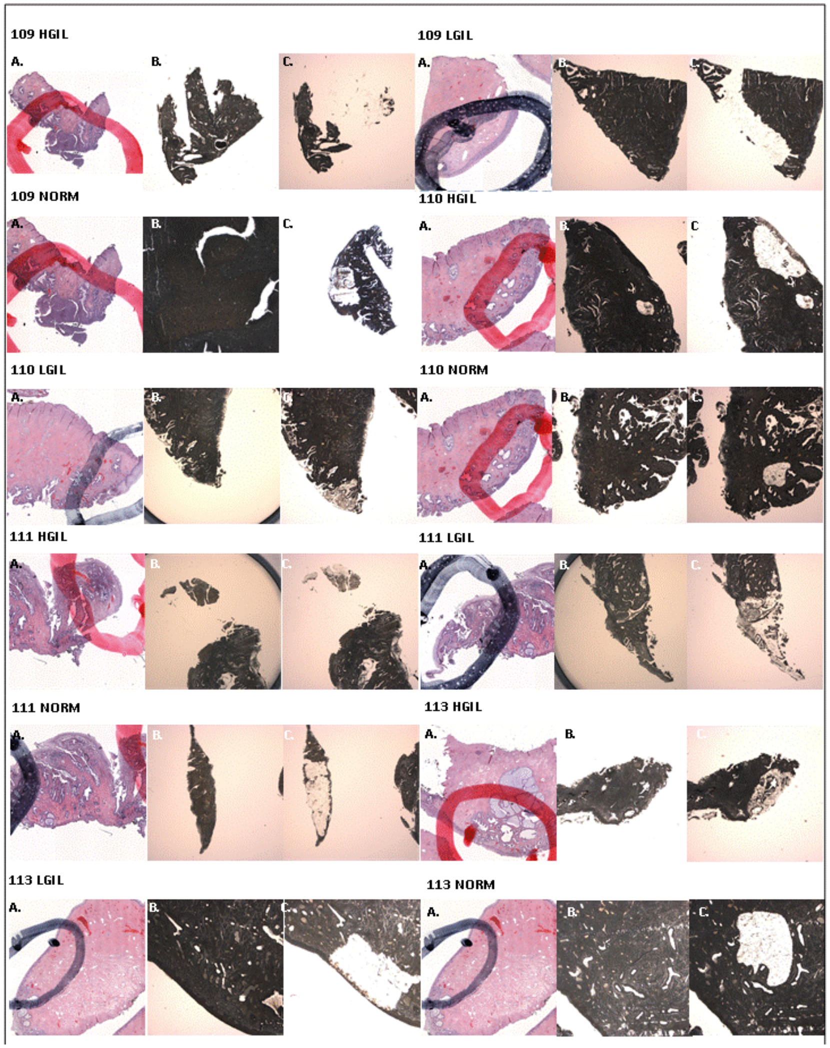

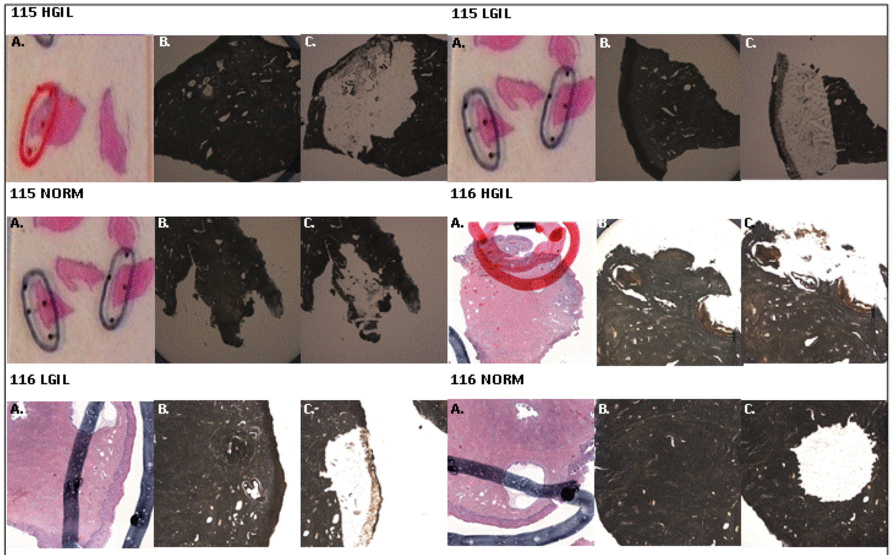

A.-Reference Slide; B.-Slide before laser capture microdissection ; C.-Slide after laser capture microdissection

HGIL-High Grade Intraepithelial Lesion; LGIL-Low Grade Intraepithelial Lesion; NORM-Adjacent Normal Tissue

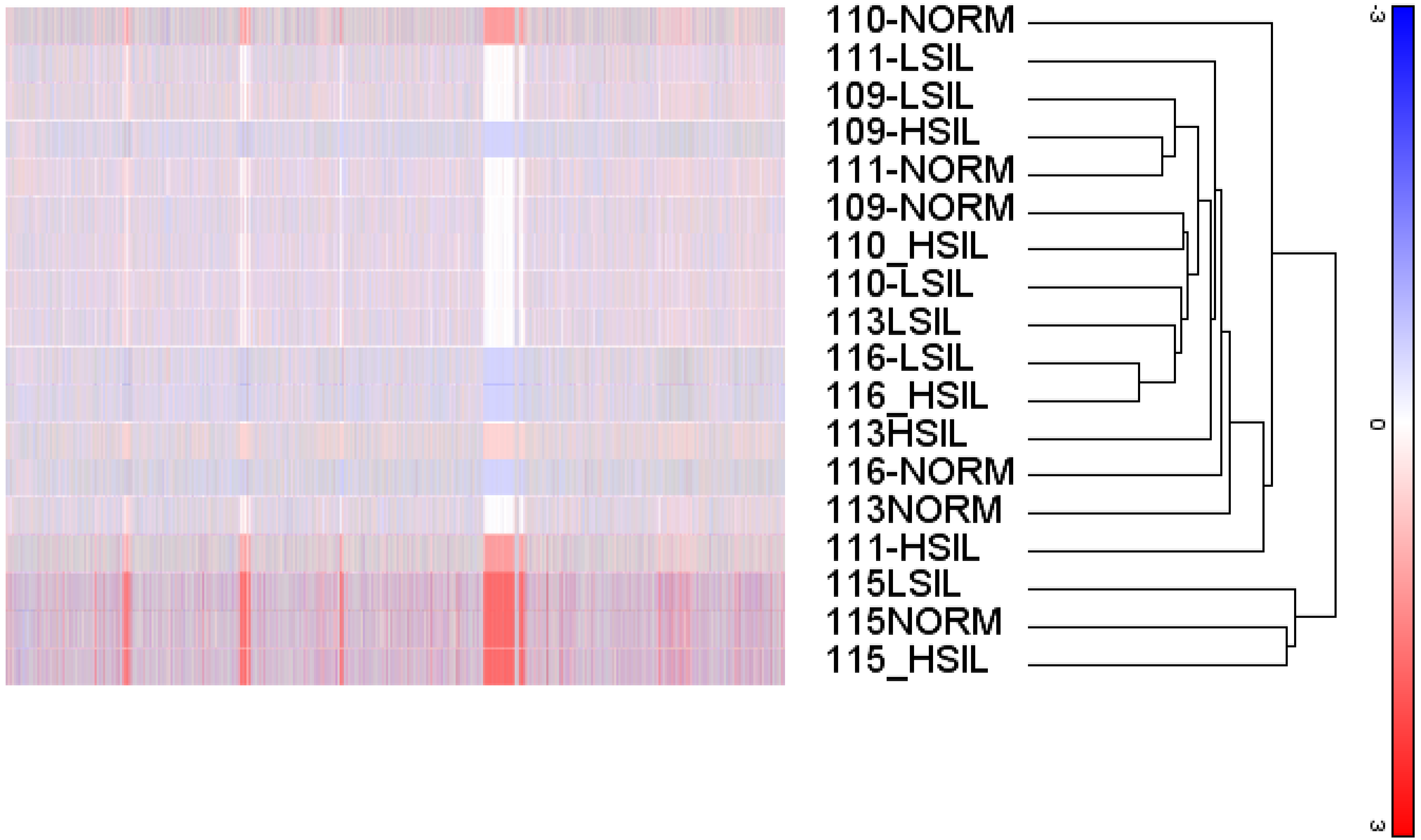
110-NORM
111-LSIL
109-LSIL
109-HSIL
111-NORM
109-NORM
110_HSIL
110-LSIL
113LSIL
116-LSIL
116_HSIL
113HSIL
116-NORM
113NORM
111-HSIL
115LSIL
115NORM
115_HSIL
-3
0
3
relative

Supplementary Figure S3

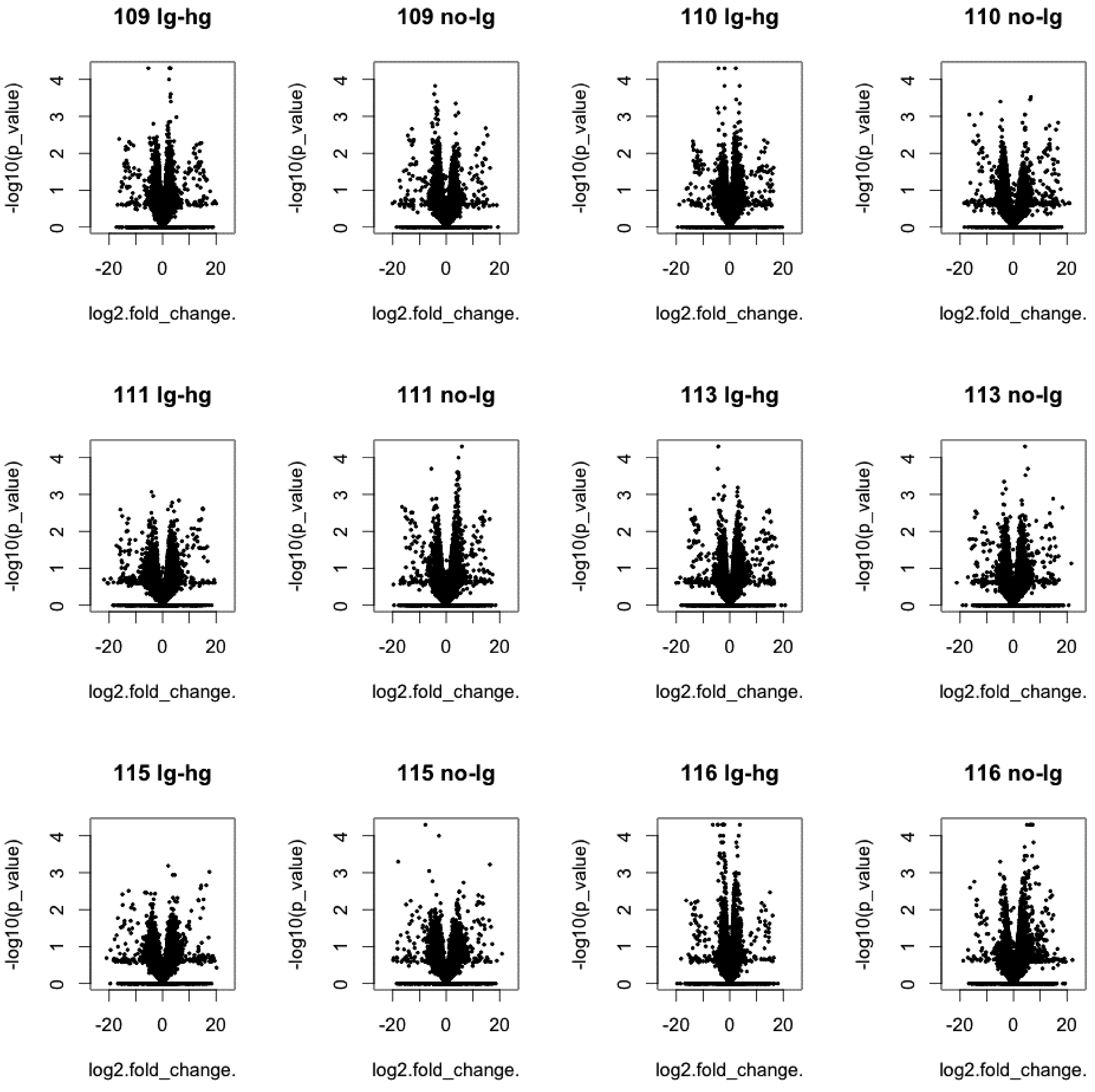

Supplemantary Figure S4A

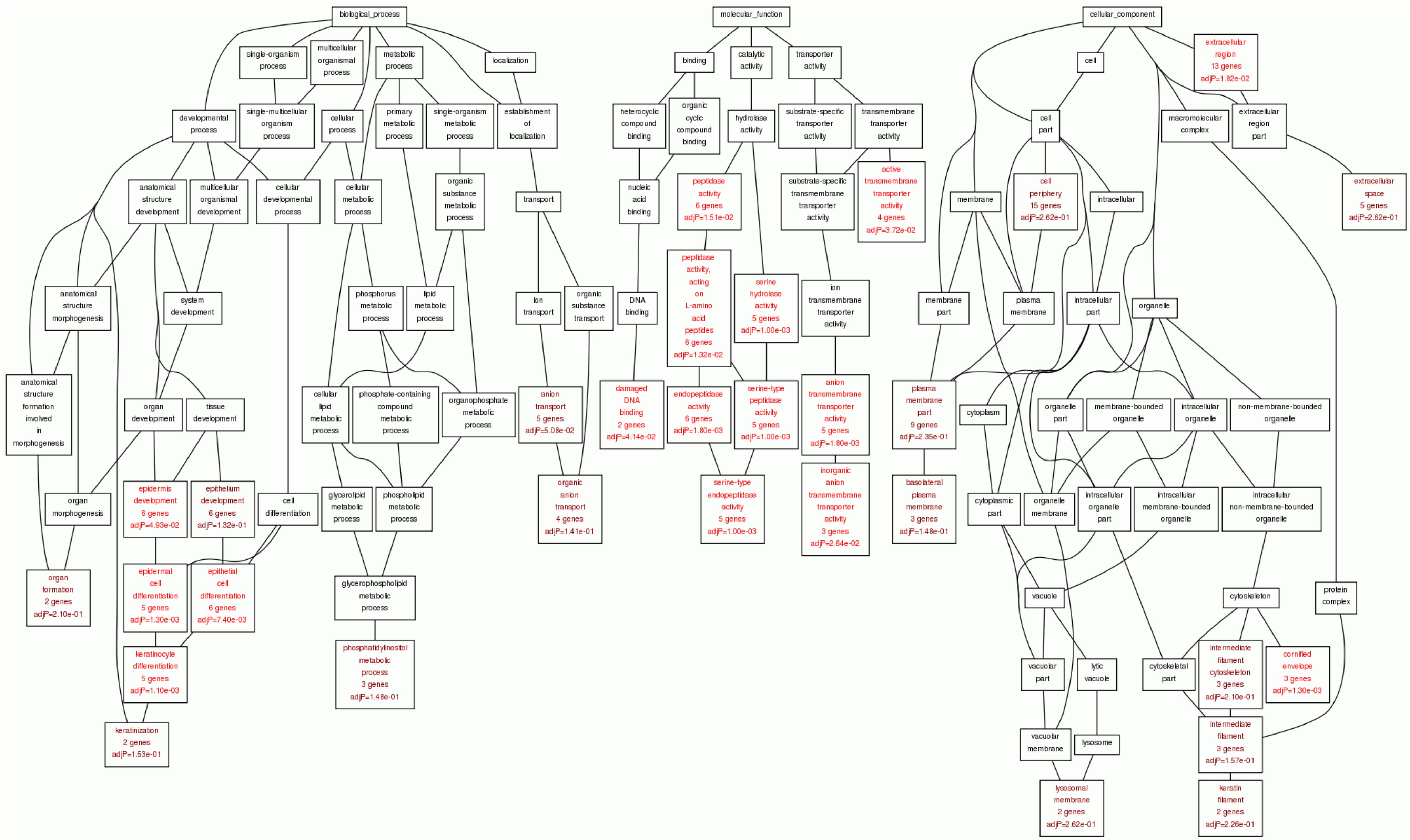

Supplementary Figure S4B

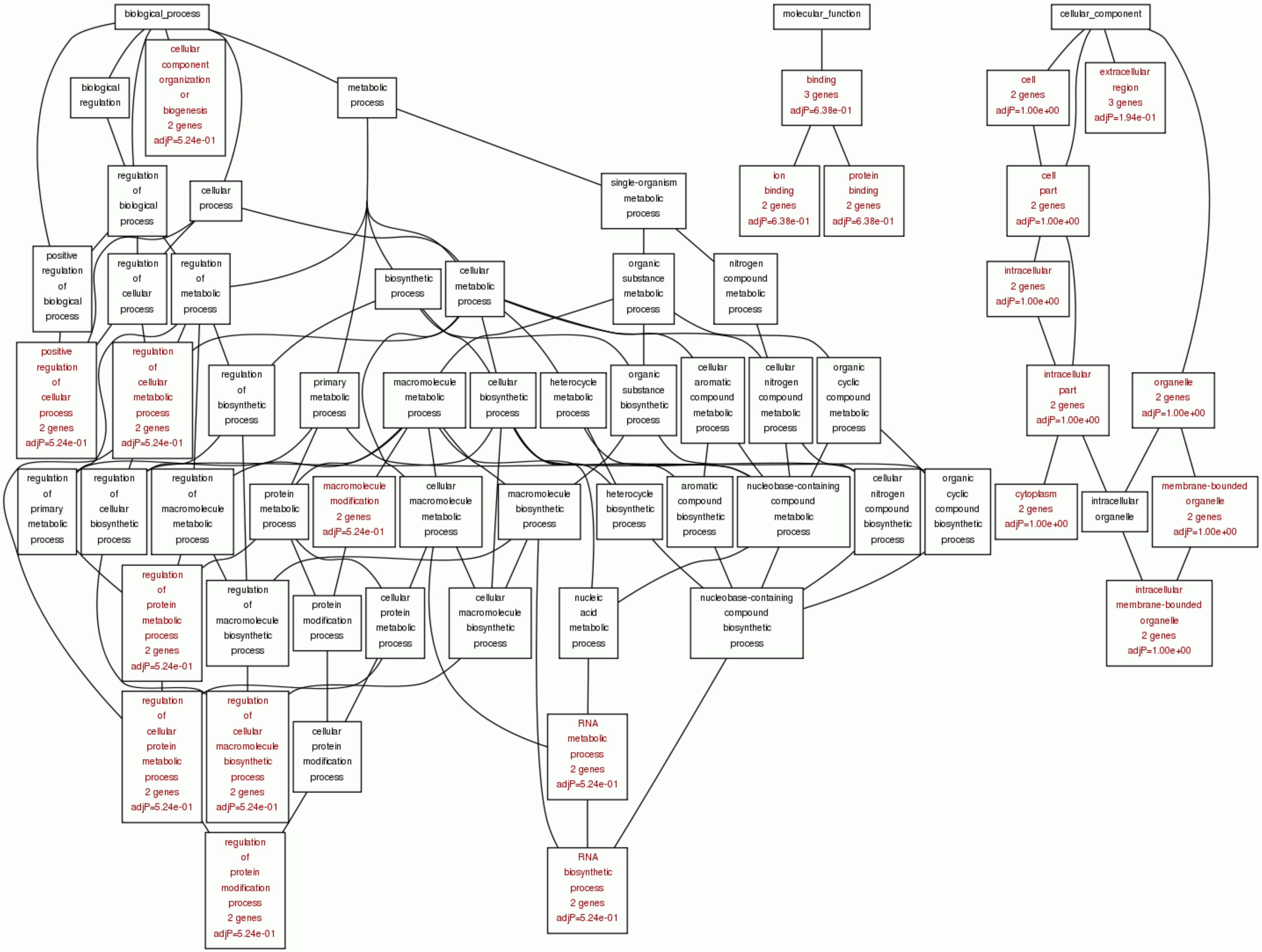

Supplement: Supplementary file 1 [file Data_Sheet_1.ZIP › Supplementary Tables and Figures.PDF]
